# Use of a Tn5-based transposon system to create a cost-effective *Zymomonas mobilis* for ethanol production from lignocelluloses

**DOI:** 10.1186/1475-2859-12-41

**Published:** 2013-05-02

**Authors:** Xi Zhang, Tianyv Wang, Wen Zhou, Xianghui Jia, Haoyong Wang

**Affiliations:** 1 Key Laboratory of Fermentation Engineering (Ministry of Education), Hubei Provincial Key Laboratory of Industrial Microbiology, College of Bioengineering, Hubei University of Technology, Wuhan, 430068, China; 2 Nanhu Middle School, Wuhan, 430060, China; 3 Zhejiang Hisun Pharmaceutical Co., Ltd., Taizhou, 318000, China

**Keywords:** *Zymomonas mobilis*, Heat shock protein, *yfdZ*, *metB*, Xylose fermentation, Ethanol production

## Abstract

**Background:**

Current methods of ethanol production from lignocelluloses generate a mixture of sugars, primarily glucose and xylose; the fermentation cells are always exposed to stresses like high temperature and low nutritional conditions that affect their growth and productivity. Stress-tolerant strains capable of using both glucose and xylose to produce ethanol with high yield are highly desirable.

**Results:**

A recombinant *Zymomonas mobilis* (*Z. mobilis)* designated as *HYMX* was constructed by integrating seven genes (*Pfu-sHSP, yfdZ, metB, xylA, xylB, tktA* and *talB*) into the genome of *Z. mobilis* CP4 *(CP4)* via Tn5 transposon in the present study. The small heat shock protein gene (*Pfu-sHSP*) from *Pyrococcus furious* (*P. furious*) was used to increase the heat-tolerance, the *yfdZ* and *metB* genes from *E. coli* were used to decrease the nutritional requirement. To overcome the bottleneck of *CP4* being unable to use pentose, xylose catabolic genes (*xylA, xylB, tktA* and *talB*) from *E. coli* were integrated into *CP4* also for construction of the xylose utilizing metabolic pathway.

**Conclusions:**

The genomic integration confers on *Z. mobilis* the ability to grow in medium containing xylose as the only carbon source, and to grow in simple chemical defined medium without addition of amino acid. The *HYMX* demonstrated not only the high tolerance to unfavorable stresses like high temperature and low nutrient, but also the capability of converting both glucose and xylose to ethanol with high yield at high temperature. What’s more, these genetic characteristics were stable up to 100 generations on nonselective medium. Although significant improvements were achieved, yeast extract is needed for ethanol production.

## Introduction

Among various ethanol-producing microbes, the gram-negative bacterium *Z. mobilis* is an efficient ethanol producer with favorable features that are at least equal to those from the more familiar brewer’s yeast [1,2]. Ethanol production at high temperature has received much attention because fermentation processes conducted at elevated temperatures will significantly reduce cooling costs and improve simultaneous saccharification, fermentation, distillation and suitability for use in tropical countries. However, the temperatures suitable for *Z. mobilis* are relatively low (25 to 32°C). *Z. mobilis* is unable to produce ethanol effectively under temperature above 38°C. Creation of the heat-tolerance strains will be of great value for the ethanol industry, while screens for recombinant *Z. mobilis* mutants able to produce ethanol efficiently at high temperature have never been performed before.

The hyperthermophilic archaeon *P. furiosus* expresses a small, α-crystallin-like protein in response to extreme temperatures above 103°C. This *s*mall heat shock protein (*Pfu-sHSP*) gives cellular protections from extremely high temperatures [3,4] like the *α-*crystallin eye lens protein, acts as molecular chaperone and prevents aggregation of denatured proteins under heat stress. It can prevent *E. coli* cellular proteins from aggregation above 100°C, and can significantly enhance the viability of mesophilic organisms such as *E. coli* under lethal temperatures [4]. The introducing of the gene *Pfu-sHSP* might be able to protect the mesophilic enzymes of *Z. mobilis* from aggregation at elevated temperatures to increase its ethanol production under heat-stress.

The growth of *Z. mobilis* usually requires complex rich medium containing yeast extract, has long been known to require lysine and methionine [5,6]. The whole-genome sequencing has revealed the specific reasons for these deficiencies. The only genes missing for lysine and methionine synthesis are *yfdZ* and *metB*, respectively [6-8]. The *yfdZ* and *metB* genes encode a PLP-dependent aminotransferase and a PLP (pyridoxal phosphate)-dependent cystathionine gamma-synthase, respectively. As the *Z. mobilis* suffered from malnutrition may not be able to maintain its normal ability to produce ethanol in low nutritional conditions which are common at the end of a batch, and rich medium will significantly increase the cost of ethanol production, *yfdZ* and *metB* genes were introduced into *Z. mobilis* in this research to verify whether or not we can get a mutant *Z. mobilis* with lower nutritional requirements.

Current methods for pretreatment of lignocelluloses for ethanol production generate a mixture of pentose (C5) and hexose (C6) sugars. It has already been proved that the heterologous xylose metabolic enzymes (xylose isomerase-*xylA*, xylulokinase-*xylB*, transaldolase-*tktA*, and transketolase-*talB*) from *E. coli* can be expressed in *Z. mobilis* to enable the bacteria to ferment xylose [9,10]. U.S. Pat. No. 5,514,583 discloses a plasmid transformed *Z. mobilis* with the ability to ferment xylose (*CP4*/pZB4 and pZB5), having plasmid vectors (pZB4 and pZB5) encoding *xylA, xylB, tktA* and *talB* exogenous genes from *E. coli*, and further comprising at least one promoter (P*GAP* and P*ENO*) recognized by *Z. mobilis* which regulates the expression of at least one of the said genes. The strain is capable of growing on xylose as the sole carbon source and fermenting xylose to ethanol at about 88% of the maximum theoretical yield. By transforming the plasmid encoding *xylA, xylB, tktA* and *talB* exogenous genes from *E. coli,* they succeeded in getting a xylose-fermenting *Zymomonas*.

To overcome the instability limitations of foreign plasmids exhibited under both selective and non-selective conditions which are generally perceived as undesirable for industrial applications, Tn5 transposition mutagenesis in *Z. mobilis* was used in this research. Tn5 transposon can mediate genome DNA rearrangements and integrations of foreign DNA via a cut-and-paste mechanism. The only macromolecular components required for this process are the transposase; the transposon, which can presumably be any sequence that is defined by two specific inverted 19 bp sequences; and the target DNA into which the insertions are made [11].

Mediated by Tn5 transposon, foreign genes were integrated into the genome of *CP4* in this study. The *Pfu-sHSP* gene was used for increasing the tolerance to high temperature; the *yfdZ and metB* genes were used for increasing the tolerance to low nutrient. Xylose catabolic genes (*xylA, xylB, tktA* and *talB)* were used for adding a new metabolic pathway for the mutate *CP4* to utilize xylose to produce ethanol. The study showed that the stable integration of seven foreign genes at the same time was possible, and the mutant *HYMX* demonstrated the capability of converting mixed sugars to ethanol with high yield, demonstrated also a high tolerance to heat stress and low nutrient stress.

## Results

### An obvious decrement in nutritional requirement

Although possesses a complete set of genes for synthesis of all amino acids, *Z. mobilis* do not have one gene in the lysine (*yfdZ*) pathway and one gene in the methionine (*metB*) pathway [6,8]. Wild type *Z. mobilis* CP4 can grow well in medium containing yeast extract, but can not grow in simple chemical medium and in medium with xylose as the sole carbon source because it has no xylose metabolic pathway.

**Figure 1:**
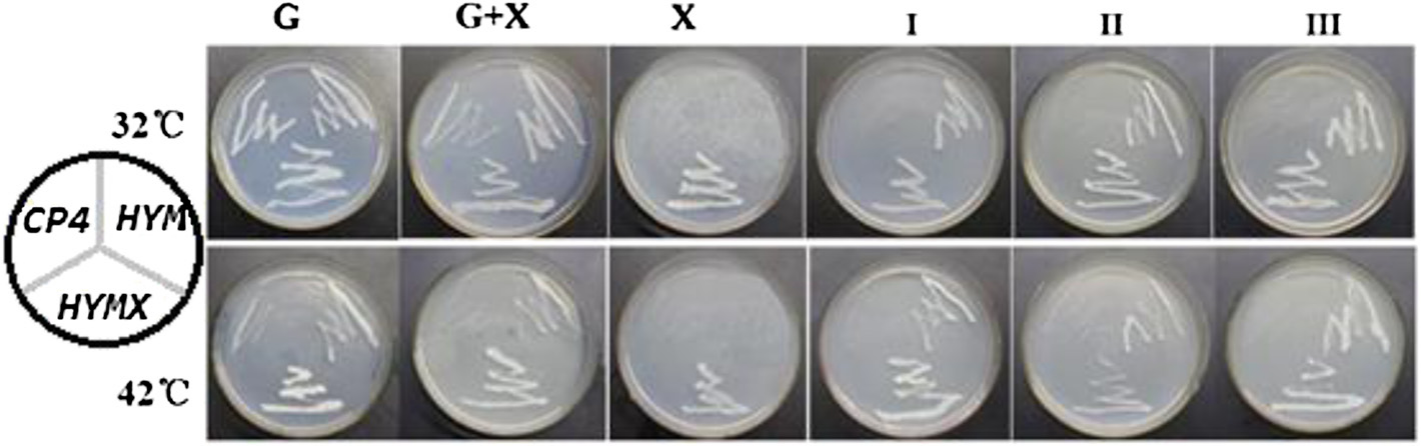
**The growth properties of *CP4, HYM *and *HYMX *on simple chemical medium supplemented with yeast extract or without yeast extract.** All strains were obtained by centrifugal sedimentation method. The same weight wet cells were washed by ultrapure water for three times before being inoculated, then all petri dishes were kept at 32°C or 42°C for 60 hours. *CP4* grows well in medium (Table 1) contain yeast extract and glucose (plate G, plate G + X); can’t grow in simple chemical medium without yeast extract (plate I, plate II and plate III) and in medium contain yeast extract with xylose as the only carbon source (plate X). *HYM* and *HYMX can* grow in simple chemical medium without yeast extract (plate I, plate II and plate III). *HYMX can* grow also in medium when xylose as the only carbon source (plate X).

With the *yfdZ* and *metB* genes, the *HYM* and *HYMX* strains showed normal growth in simple chemical medium both at 32°C and 42°C. With the xylose catabolic genes (*xylA, xylB, tktA* and *talB)* together with *yfdZ* and *metB* genes from *E. coli, HYMX* strain showed normal growth in medium when xylose was the sole carbon source. *HYMX* could grow in simple chemical defined medium, as well as in medium when xylose was the sole carbon source. In contrast, neither in simple chemical defined medium nor in medium when xylose as the sole carbon source, could *CP4* grow (Figure 1, Table 1).

**Table 1:** Different raw materials used for creation of the experimental growth medium (g/L)

**Plate name**	**G**	**G + X**	**X**	**I**	**II**	**III**
Yeast extract	10.0	10.0	10.0	0	0	0
KH_2_PO_4_	1.0	1.0	1.0	1.0	1.0	1.0
MgSO_4_ · 6H_2_O	1.0	1.0	1.0	0	0	0
Carbamide	1.0	1.0	1.0	1.0	0	1.0
Glucose	10.0	5.0	0	10.0	10.0	10.0
NH_4_Cl	0	0	0	0	10.0	0
MgCl_2_ · 6H_2_O	0	0	0	0	1.0	1.0
Xylose	0	5.0	5.0	0	0	0
NaCl	0	0	0	0	8.0	8.0
CaCl_2_ · .2H_2_O	0	0	0	0	0.4	0.4
KCl	0	0	0	0	1.0	1.0
Agar	20.0	20.0	20.0	20.0	20.0	20.0

**Figure 2:**
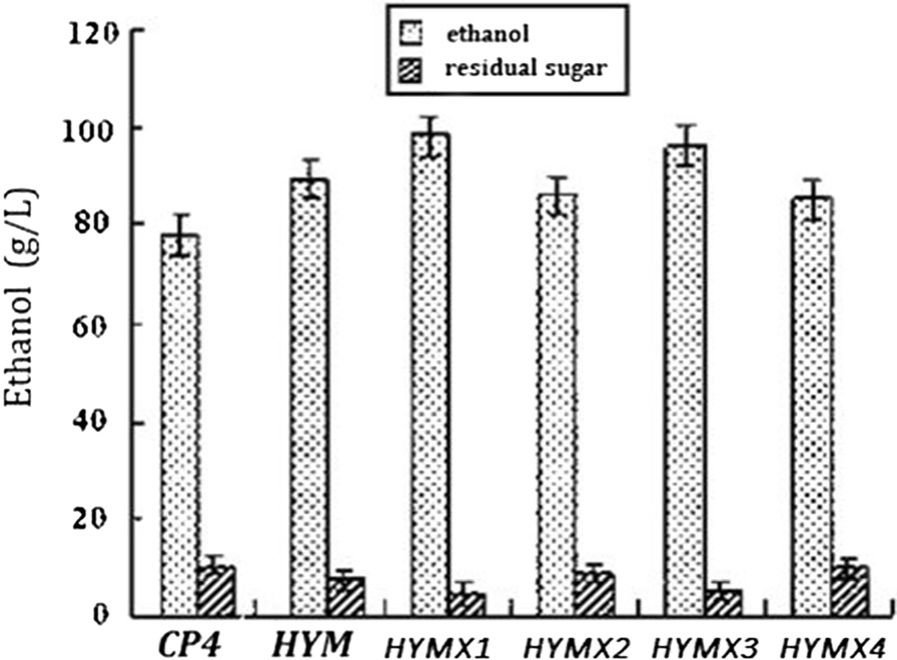
**Comparisons of ethanol production and residual sugar between three different kinds of *Z. mobilis *strains.** Cultures were grown in RM medium (10 g/L yeast extract, 1 g/L KH_2_PO_4_, 1 g/L Carbamide, 0.5 g/L MgSO_4_.6H_2_O) with 230 g/L glucose as carbon source under anaerobic conditions at 32°C. All strains were tested by three parallel experiments. After 60 hours fermentation, the highest ethanol concentration of *HYM* and *HYMX* was 87.1 g/L, 98.7 g/L, respectively, while the original strain *CP4* was 78.4 g/L.

**Figure 3:**
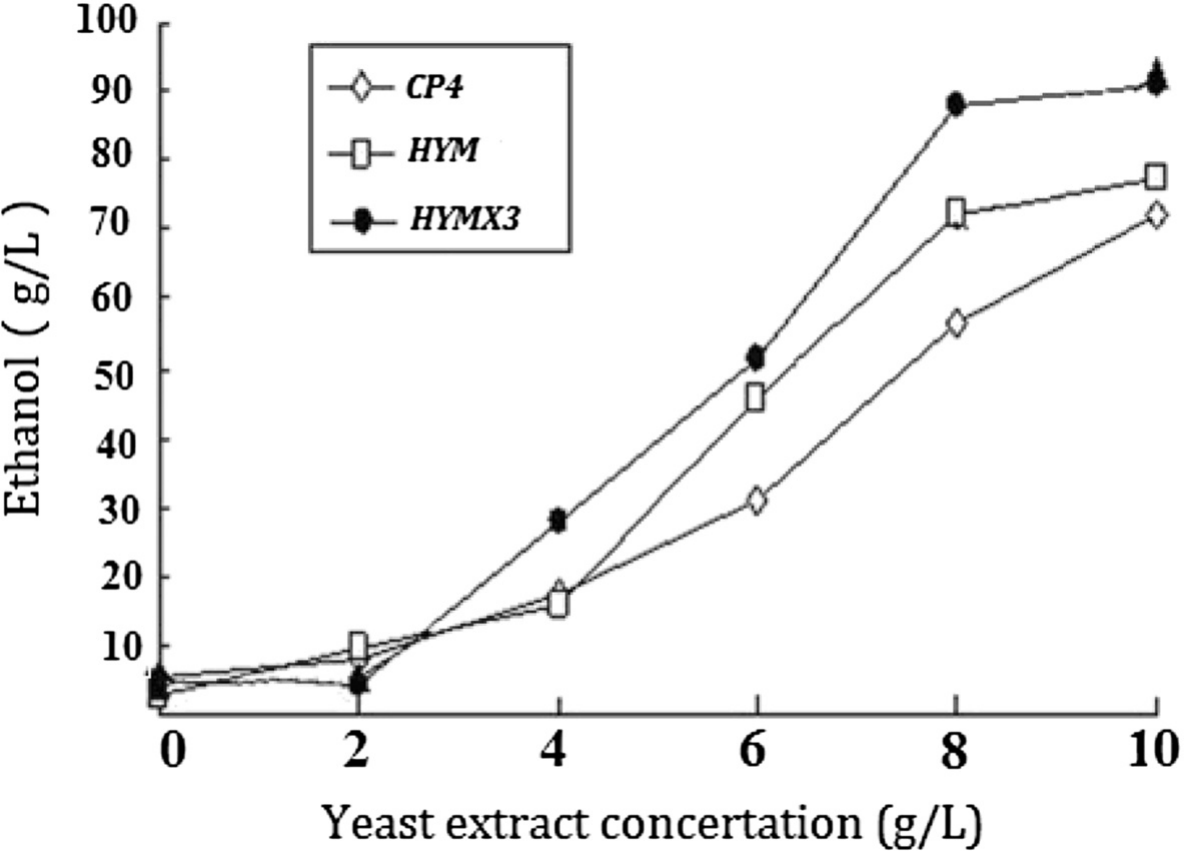
**Comparisons of ethanol production between three kinds of *Z. mobilis *strains in medium with different concentration of yeast extract.** Strains were grown in medium (1 g/L KH_2_PO_4_, 1 g/L Carbamide, 0.5 g/L MgSO_4_.6H_2_O) with 230 g/L glucose as carbon source, supplemented with different amounts of yeast extract (as indicated) under anaerobic conditions at 32°C. All strains were tested by three parallel experiments. After 60 hours fermentation, ethanol production of all strains was measured immediately by GC.

When cultured in RM medium (10 g/L yeast extract, 1 g/L KH_2_PO_4_, 1 g/L Carbamide, 0.5 g/L MgSO_4_.6H_2_O) with 230 g/L glucose as carbon source. After 60 hours fermentation, the ethanol concentration of *HYMX3* was 98.7 g/L (theoretical yield 83.5%), while the ethanol concentrations of *HYM* and *CP4* was 87.1 g/L and 78. 4 g/L, respectively (Figure 2). The effects of yeast extract on ethanol production of three kinds of *Z. mobilis* strains were studied also in this research. In medium (1 g/L KH_2_PO_4_, 1 g/L Carbamide, 0.5 g/L MgSO_4_.6H_2_O) with 230 g/L glucose as carbon source under anaerobic conditions at 32°C, when the yeast extract concentration changed from 0 to 4 g/L, there were no obviously differences of ethanol yield between 3 strains. When the yeast extraction concentration changed from 4 g/L to 8 g/L, after 60 hours fermentation, the ethanol concentration of *HYMX3*, *HYM* and *CP4* was increased by 60.4 g/L, 54.5 g/L and 39.6 g/L, respectively. From the trend lines, we can deduce that in low nutrient medium, *HYMX*3 was more effective to produce ethanol than others (Figure 3).

### High yield fermentation of xylose and glucose

Theoretically, 100 grams of glucose will produce 51.4 grams of ethanol and 48.6 grams of carbon dioxide. Xylose is the second most abundant carbohydrate in nature, the theoretical yield of ethanol from xylose is defined as 51.1 grams of ethanol per 100 grams of xylose (5 mol of ethanol/3 mol of xylose). However, in practice, the microorganisms use some of the sugar for growth and the actual yield is less than 100%.

From all the mutants developed, four *XU* strains and four *HYMX* strains were chosen for further fermentation research with *CP4* as a control. Strains *XU* have stable insertion of 4 foreign structural genes (*xylA/xylB* and *talB/tktA* from *E. coli*) and strains *HYMX* have stable insertion of 7 foreign structural genes (*xylA*, *xylB*, *talB*, *tktA*, *metB*, *yfdZ* from *E. coli* and *Pfu-sHSP* from *P. furiosus*) into the *Z. mobilis CP4* genome.

**Figure 4:**
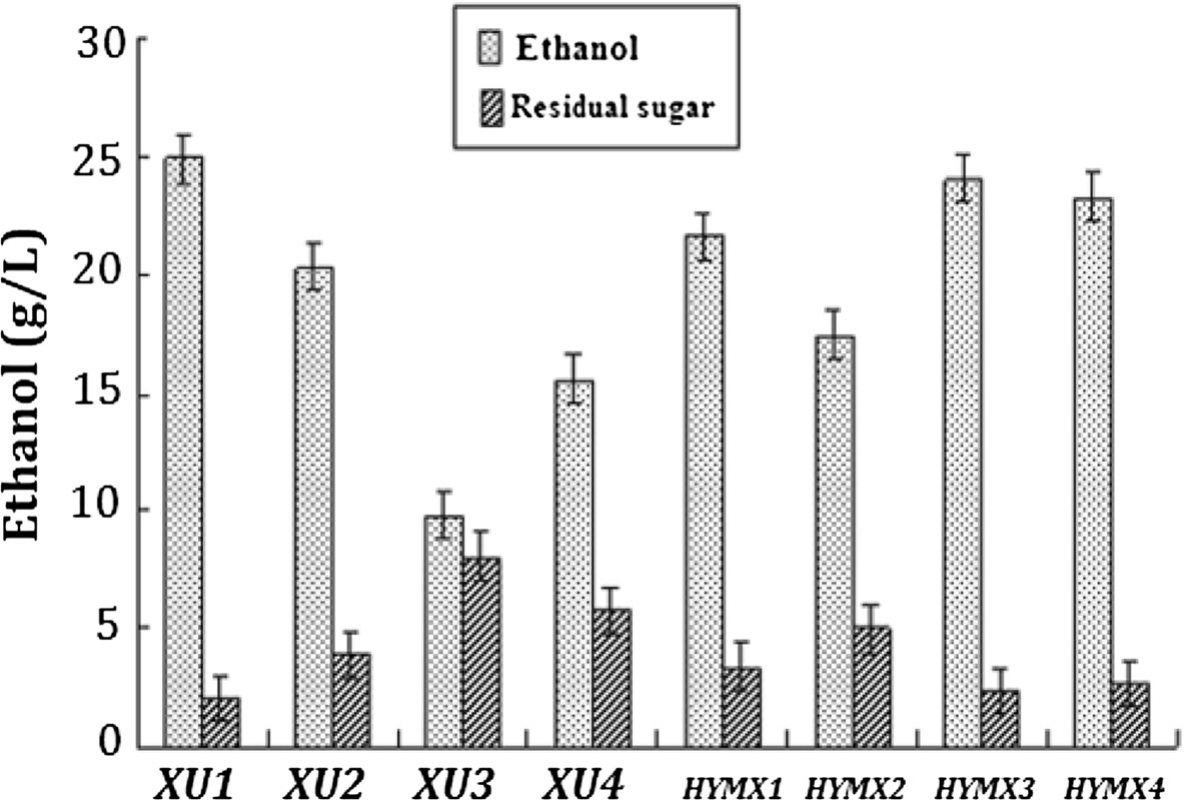
**Comparisons of ethanol production and residual sugars of *Z. mobilis *strains in medium when xylose was the only carbon resource.** Cultures were grown in RM medium (10 g/L yeast extract, 1 g/L KH_2_PO_4_, 1 g/L Carbamide, 0.5 g/L MgSO_4_.6H_2_O) with 60 g/L xylose as the only carbon source under anaerobic conditions at 32°C. All strains were tested by three parallel experiments. After 60 hours fermentation, ethanol production of all strains was measured immediately by GC. The highest ethanol concentration of *XU1* and *HYMX3* was 24.9 g/L, 24.1 g/L, respectively.

**Figure 5:**
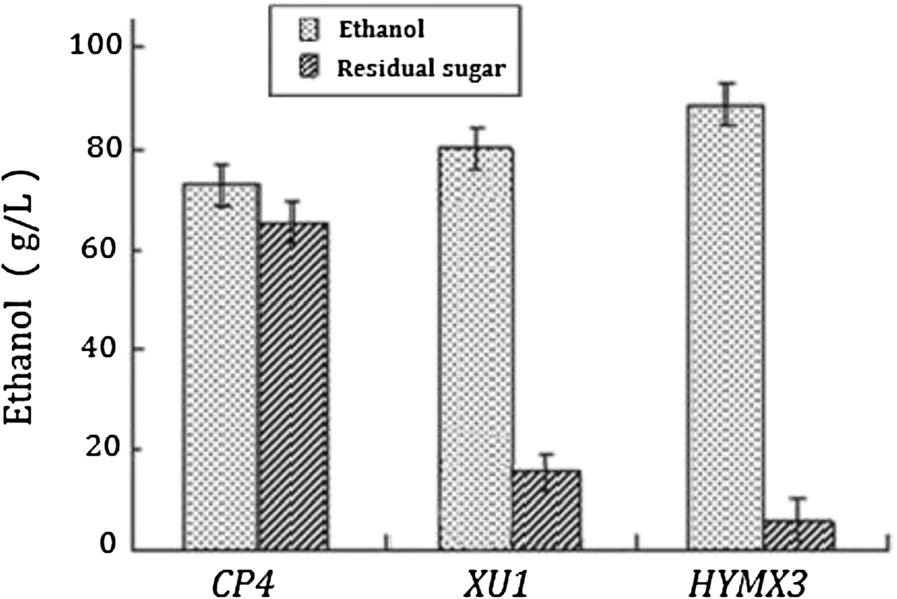
**Comparisons of ethanol production and residual sugars between three kinds of *Z. mobilis *strains in medium containing mixed sugars.** Cultures were grown in RM medium (10 g/L yeast extract, 1 g/L KH_2_PO_4_, 1 g/L Carbamide, 0.5 g/L MgSO_4_.6H_2_O) with 170 g/L glucose and 60 g/L xylose as carbon source anaerobically at 32°C. All strains were tested by three parallel experiments. After 60 hours anaerobic fermentation, the average ethanol concentration of *CP4*, *XU1* and *HYMX3* was 72.7 g/L, 83.8 g/L and 88.9 g/L, respectively.

*Z. mobilis* CP4 can not grow in the medium (10 g/L yeast extract, 1 g/L KH_2_PO_4_, 1 g/L Carbamide, 0.5 g/L MgSO_4_.6H_2_O) containing xylose as the sole carbon source, while the recombinant strains *XU* (1 ~ 4) and *HYMX* (1 ~ 4) can grow and produce ethanol with high yield. As was shown in Figure 4 when xylose (60 g/L) was the only carbon source, *HYMX* (1 ~ 4) and *XU* (1 ~ 4) were able to ferment xylose to produce ethanol. After 60 hours fermentation, the highest ethanol concentration of *XU (1 ~ 4)* was 24.1 g/L (theoretical yield 78.6%). The highest ethanol concentration of *HYMX* (1 ~ 4) was 24.9 g/L (theoretical yield 81.2%). In the mixed sugar medium (containing 170 g/L glucose and 60 g/L xylose) after 60 hours fermentation, the average ethanol concentration of *CP4* was 72.7 g/L (theoretical yield 61.6%); the average ethanol concentration of *XU1* was 83. 8 g/L (theoretical yield 71.0%), which was 11.5% higher than *CP4.* While the average ethanol concentration of *HYMX3* was 88.9 g/L (theoretical yield 75.3%), which was 22.3% higher than *CP4* (Figure 5).

These results clearly demonstrated that the integration of *metB, yfdZ* and *Pfu-sHSP* confers on the *Z. mobilis* the ability to produce more ethanol, and that the integration of *xylA/xylB* and *talB/tktA* succeeded in giving a xylose-fermenting *Zymomonas*.

### Significant increasement in ethanol yield at high temperature

As the *Pfu-sHSP* from *P. furiosus* could potentially be used to maintain cell viability under unfavorable conditions such as heat shock or chemical treatments, fermentation experiments were performed to compare the fermentation performance of the engineered strains with *CP4* at high temperature in the mixed sugar medium (10 g/L yeast extract, 1 g/L KH_2_PO_4_, 1 g/L Carbamide, 0.5 g/L MgSO_4_.6H_2_O) containing 170 g/L glucose and 60 g/L xylose. No obvious difference of ethanol production was observed between the mutant and the wild-type *Z. mobilis,* when fermentation was carried out at normal temperature at 32°C. Under high temperature up to 40°C, the stable integration of *Pfu-sHSP* showed a significant impact on the ethanol production of *Z. mobilis* from glucose, xylose or mixed sugar. The *Pfu-sHSP* bearing strain *HYMX3* and *HYM* were found to be more potent to produce ethanol than the wild type *CP4*.

**Figure 6:**
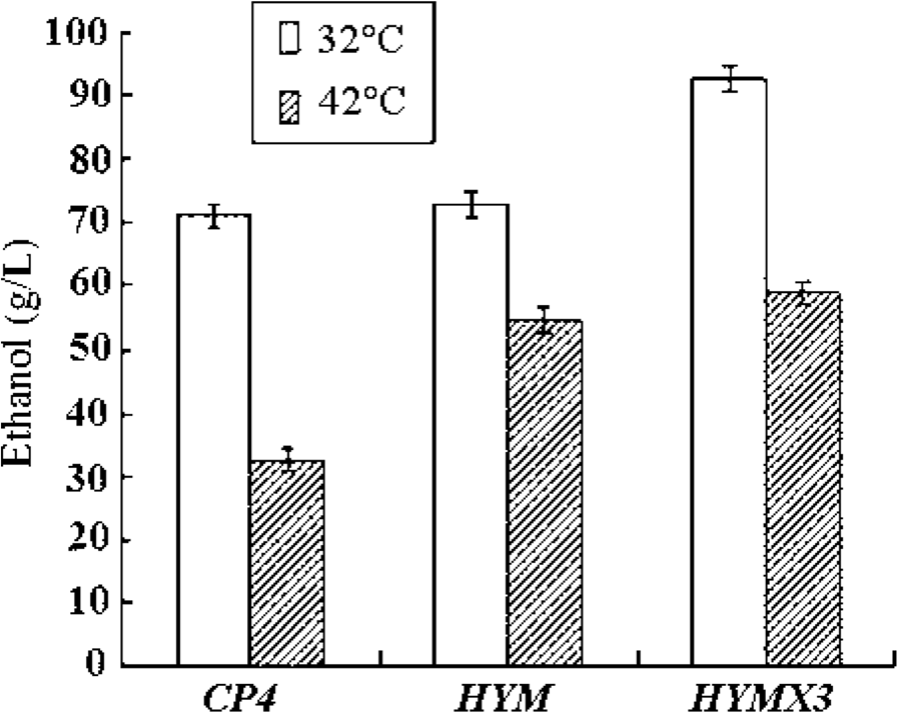
**Comparisons of ethanol production between three kinds of *Z. mobilis *strains at different fermentation temperature.** Cultures were grown in RM medium (10 g/L yeast extract, 1 g/L KH_2_PO_4_, 1 g/L Carbamide, 0.5 g/L MgSO_4_.6H_2_O) with 170 g/L glucose and 60 g/L xylose as carbon source at 32°C or 42°C. All strains were tested by three parallel experiments. When the temperature was up to 42°C from 32°C, after 60 hours anaerobic fermentation, the average ethanol production of *CP4*, *HYM* and *HYMX* was reduced by 47%, 28% and 31%, respectively. However, the ethanol production of *HYMX3* was still the highest.

For *Z. mobilis* strains without *Pfu-sHSP,* no matter can or cannot use xylose, their average ethanol production decreased more significantly when the temperature was up to 40°C from 32°C. After 60 hours fermentation,the ethanol production of *CP4, HYM* and *HYMX* was reduced by 47%, 28% and 31%, respectively. With the *Pfu-sHSP* gene, *HYMX3* and *HYM* demonstrated a significantly improved tolerance to high temperature (Figure 6).

### Stability test

Two kinds of chromosomal integrated strains of *Z. mobilis* capable of using xylose were engineered in this research. After preliminary evaluation, strain *XU1* and *HYMX3* were selected for further stability test and fermentation studies.

Frozen stock culture was transferred into medium (10 g/L yeast extract, 1 g/L KH_2_PO_4_, 1 g/L Carbamide, 0.5 g/L MgSO_4_.6H_2_O) with 90 g/L glucose and 10 g/L xylose (RMGX). Overnight grown culture was transferred into an RMGX tube containing 10 mL of medium with no antibiotic to an initial OD of 0.02 at 600 nm. The inoculum culture was incubated on a rotary shaker at 150 rpm 32°C for 16 hours or until an OD of 2 to 3 at 600 nm was reached. At the end of 100 generations, the grown culture was tested for fermentation of glucose or xylose, and co-fermentation of glucose with xylose in a 100-mL shake flask containing 80 mL medium (10 g/L yeast extract, 1 g/L KH_2_PO_4_, 1 g/L Carbamide, 0.5 g/L MgSO_4_.6H_2_O, 170 g/L glucose, 60 g/L xylose). *XU1* and *HYMX3* could ferment glucose and xylose, could co-ferment glucose and xylose to produce ethanol with high yield, with no obvious differences than they were first been isolated. The PCR reactions also confirmed that 2 strains were stable up to 100 generations on nonselective medium.

## Discussions

Ethanol production from renewable resources, such as biomass, is a promising alternative to compete with and eventually replace nonrenewable fossil fuels; has considerable advantages in terms of sustainability, lower greenhouse gas emissions, and cost reduction. Several groups have tried to develop ethanologenic gram-positive bacteria, but with limited success . However, the gram-negative bacterium *Z. mobilis* is an efficient ethanol producer with favorable features that are at least equal to those from the more familiar yeast. With the recent advances in biotechnology, *Z. mobilis* has the potential to play a key role in making production of ethanol much more economical.

In nature, *Z. mobilis* is found in high sugar content solutions fermenting sugars to ethanol in a high productivity process. It has several advantages over yeast. Ethanol yield of *Z. mobilis* is as high as 97% of theoretical yield when glucose or fructose is the substrate. It has a high specific ethanol productivity which is up to 2.5 times higher than yeast [12]. It can tolerate up to 400 g/L glucose, up to 160 g/L ethanol and up to 8 g/L acetic acid (at pH 6) [13]. Its ability to grow at lower pH (5.0-5.5) helps to reduce the aseptic requirements for fermentation, thus rendering the fermentation process more economical.

The growth of *Z. mobilis* has long been known to require lysine, methionine, and the whole-genome sequencing had revealed the specific reasons for these deficiencies. The only genes missing for lysine and methionine synthesis are *yfdZ* and *metB*, respectively. Herein for the first time, the genome integration of the bi-cistronic *yfdZ* and *metB* genes from *E. coli* leaded to a variant of *Z. mobilis* strain can grow in simple chemical defined medium without addition of amino acid.

*Pfu-sHSP* is a kind of α-crystallin homologues proteins can act as molecular chaperones to prevent membrane destabilization or aggregation of denatured proteins under heat and other stress. Therefore it can potentially be used to maintain cell viability under unfavorable conditions, such as heat shock or chemical treatments. The *Pfu-sHSP* gene containing bacteria showed an significantly improved tolerance to high temperature in this research.

Several factors prevent the commercial usage of *Z. mobilis* in cellulosic ethanol production. The foremost hurdle is that its substrate range is limited to glucose, fructose and sucrose. Wild-type *Z. mobilis* cannot ferment C5 sugars like xylose which are important components of lignocellulosic hydrolysates. National Renewable Energy Laboratory (NREL) has made significant contributions in expanding its substrate range by introducing xylose metabolizing genes (*xylA, xylB, tktA* and *talB*) from *E. coli* into *Z. mobilis* to enable the bacteria to ferment xylose [14]. This technique succeeded in giving a xylose-fermenting *Zymomonas*. Here, we demonstrated a unique strategy to allow efficient co-fermentation of glucose and xylose by *Z. mobilis*. A strong promoter (fusion of 2 strong constitutive promoters of *Z. mobilis*) was cloned to facilitate the higher level expression of genes under its control. Under the control of this strong promoter, the over expression of xylose utilization pathway seems to work efficiently. Our research results showed that the ethanol yield of the mutant *Z. mobilis* HYMX3 is as high as 0.415 g per gram of xylose when 60 g/L xylose is the only carbon source, corresponding to 81.2% of theoretical yield.

Researchers usually use shuttle plasmid to transform bacteria. Although plasmids may be readily maintained in *Z. mobilis* when cultivated in mono-culture under controlled conditions, they frequently become unstable when grown in the absence of antibiotic selection pressure. Instability may be exacerbated when *Z. mobilis* has to compete with other organisms in a mixed culture. In addition, antibiotic usage for plasmid maintenance is generally perceived as undesirable for industrial application. Thus, it is preferable to integrate the cloned DNA into the *Z. mobilis* genome where they are maintained at a low natural copy number and are thus not over-expressed, and where, at least theoretically, they should be as stable as genome DNA.

As low-cost industry fermentations may always be carried out in rather poor, toxic, viscous, nutrient-limited medium, successful fermentations from lignocelluloses to produce ethanol using *Z. mobilis* require not only the capability to convert mixed sugars to ethanol with high yield, but also the tolerance to stresses such as high temperature and low nutritional condition. These cellular characteristics are important, because current methods for pretreatment of lignocelluloses generate a mixture of sugars, primarily glucose and xylose; and because high temperature in summer and low nutritional concentration at the end of a batch are common in the ethanol industry.

## Conclusions

In this research, seven foreign genes (*yfdZ, metB, xylA, xylB, tktA* and *talB* from *E. coli*, *Pfu-sHSP* from *P. furiosus*) were integrated into the genome DNA of *CP4* to create the *Z. mobilis* HYMX3 through Tn5 transposon mediated transposition. The recombinant strain *HYMX3* has the following characteristics listed bellow at the same time: can grow in simple chemical defined medium without adding any kinds of amino acid; can ferment both glucose and xylose to produce ethanol with high productivity; having high tolerance to high temperature and low nutrient; the fermentation characteristics were stable up to 100 generations on nonselective medium. Although significant improvements were achieved, yeast extract is needed for ethanol production.

Our results demonstrated that *HYMX3* can be an alternative bio-ethanol producer for ethanol production from lignocelluloses which is much less sensitive to temperature and nutrition changes.

## Materials and methods

### Bacterial strains and cultural conditions

The bacterial strains and plasmids used are listed in Table 2. The *E. coli* K12 MG1655 was from the American Type Culture Collection (ATCC), was grown on Luria- Bertani (LB). *P. furious* strain was from ATCC, its genomic DNA was used as template for PCR amplification of the *Pfu-sHSP* gene. *Z. mobilis* ATCC 31821 (*CP4)* from ATCC was used as the template for PCR amplification of the promoter of glycelaldehyde- 3-phosphate dehdyrogenase (P*GAP*) and enolase (P*ENO*) [5].

**Table 2:** Bacterial strains and plasmids used in this study

**Strains**	**Containing genes**	**source**
*E. coli* K12 MG1655	*xylA, xylB, tktA, talB,yfdZ,metB*	ATCC 47076
*P. furious*	*Pfu-sHSP*	ATCC 43587
*Z. mobilis* CP4(*CP4*)	Wild-type *Z. mobilis*	ATCC 31821
*Z. mobilis* XU(*XU*)	*xylA, xylB, tktA, talB*	This study
*Z. mobilis* HYM(*HYM*)	*yfdZ, metB, Pfu-sHSP*	This study
*Z. mobilis* HYMX (*HYMX*)	*yfdZ, metB, Pfu-sHSP, xylA, xylB, tktA, talB*	This study
**Plasmids**		
pTN-HMY	*Pfu-sHSP,metB, yfdz*	This study
pTN-XU	*xylA, xylB, tktA, talB*	This study
pTN -HMYX	*yfdZ, metB, Pfu-sHSP, xylA, xylB, tktA, talB*	This study

*yfdZ* and *metB* genes were used for self-synthesizing lysine and methionine;

*xylA, xylB, tktA* and *talB* genes were used for utilizing xylose to produce ethanol;

*Pfu-sHSP* gene was used for increasing the tolerance to heat stresses.

*Z. mobilis* strains were cultivated anaerobically in RM medium (10 g/L yeast extract, 1 g/L KH_2_PO_4_, 1 g/L Carbamide, 0.5 g/L MgSO_4_.6H_2_O) at 32°C, supplemented with different amounts of glucose or xylose (as indicated) as carbon source. For the inoculum preparation, a single colony was added to a test tube containing 5 mL RM broth and cultured aerobically at 32°C until it reached late exponential or early stationary phase. A 1/100 dilution was added into the pre-warmed RM broth (10 mL culture into 1000 mL RM), which was then cultured aerobically at 32°C with shaking at 100 rpm for approximately 60 h. The optical density was measured with a spectrophotometer at 600 nm. Fermentation medium and fermentation products from filter-sterilized cell-free spent medium were compositionally analyzed by gas chromatography for ethanol determinations. The reducing sugar concentration was determined using the DNS method [15].

### Gas chromatography (GC)

Ethanol concentration in the medium supernatant was determined by flame ionization gas chromatography. Culture samples (1 mL) and standards were prepared by filtration. The samples and standards were quantified by injecting 1 μL of each into a model 6890 Agilent Technologies, equipped with a DB-FFAP 30 m × 0.53 mm × 1.5 μm film thickness capillary column (Agilent, Santa Clara, CA). The column operated with an initial temperature of 80°C and ramping 10°C to a final temperature of 180°C, while detector was at 250°C and injector temperature was 200°C with a post-injection dwell time of one minute. The carrier gas was N_2_ at a constant flow rate of 5 mL/min.

### Construction of Tn5 transposon vector and transposome preparation

Promoter P*GAP/ENO* was a fusion of 2 strong constitutive promoters of *Z. mobilis,* promoter glyceraldehyde-3-phosphate dehydrogenase (P*GAP*) and promoter 2-phosphoglycerate dehydratase (P*ENO*). Promoter P*GAP* was amplified from *Z. mobilis* chromosomal DNA with the primer pair: 5′ ctcgagGTTCGATCAACAACCCGAATCCTA and 5′ ggatccCTAACTTATTAAGTAGCTATTATATTC; Promoter P*ENO* was amplified with the primer pair: 5′ agatctCTCCAGTTACTCAATACGTAAC and 5′ gaattcAACCTTTCTTAAAATCTTTTAGACG. After the P*GAP* DNA was digested with BamHI and the P*ENO* DNA was digested with BglII,two DNA fragments were mixed, heated at 72°C for 60 min, and incubated with T4 DNA ligase for an additional 12 hours at 18°C to form the fusion P*GAP/ENO* promoter. The fusion promoter was PCR amplified with primer: 5′ ctcgagGTTCGATCAACAACCCGAATCCTA and 5′ gaattcAACCTTTCTTAAAATCTTTTAGACG.

**Figure 7:**
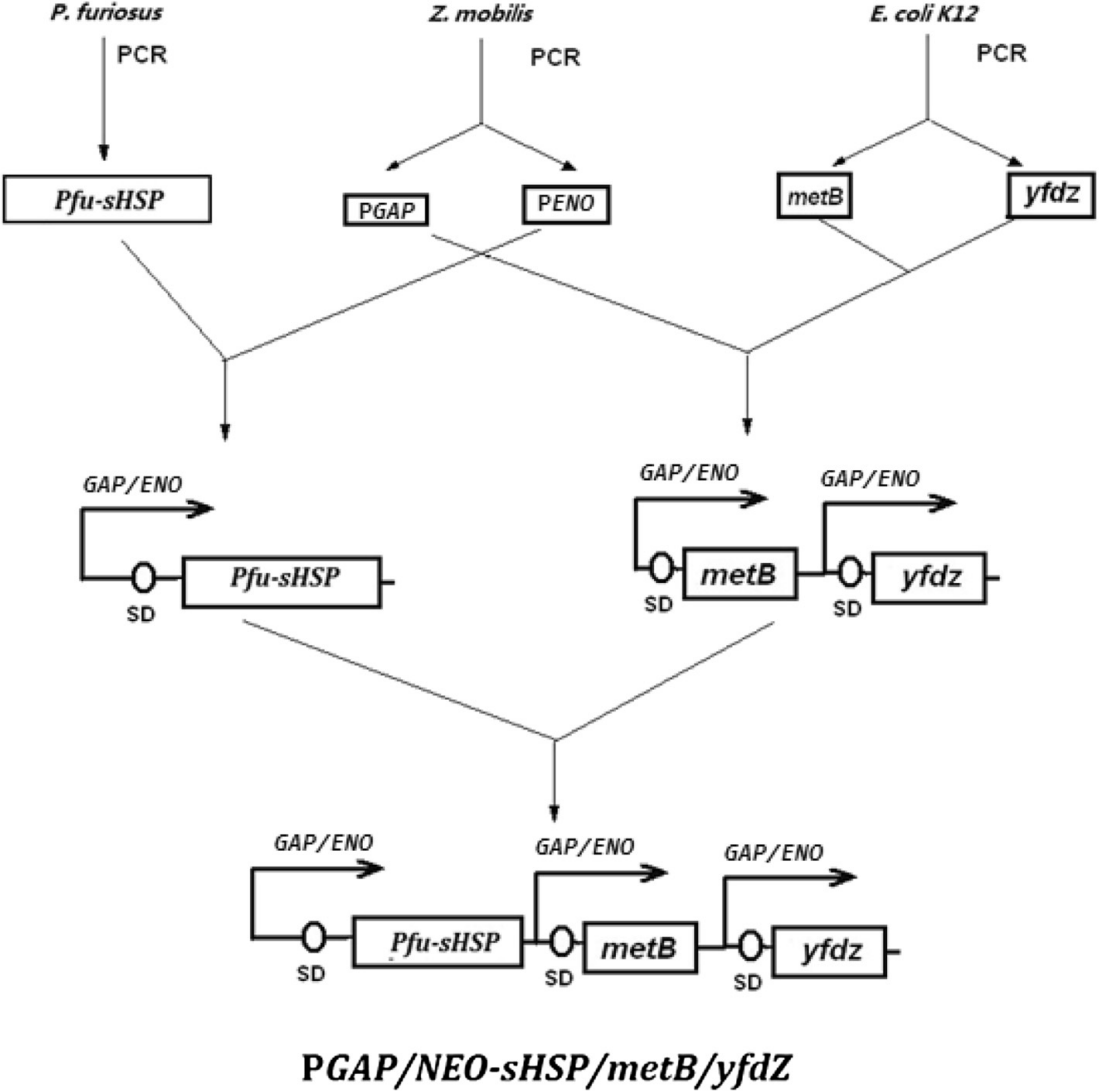
**Constructions of P*GAP/NEO-sHSP/metB/yfdZ*.** A fusion promoter (fusion of 2 strong constitutive promotors: P*GAP* and P*NEO* from *Z. mobils*) was cloned to facilitate the higher level expression of genes under its control. The *yfdZ* and *metB* genes were used to decrease nutritional requirements. The *Pfu-sHSP* gene was used to increase resistance to heat stress.

The *yfdZ* gene was amplified from *E. coli* K-12 chromosomal with the primer pair: 5′ gaattcATGGCTGACACTCGCCCTGAACGT and 5′ gtcgacTTATTCCGCGTTTTCGTGAATATG. After the digestion by EcoRI, this 1.3 kb DNA fragment was ligated with the EcoRI fragment of P*GAP/ENO* to form the *Z. mobilis* expressible P*GAP/ENO*-*yfdZ* operon. The *metB* gene was amplified from *E. coli* K-12 chromosomal with the primer pair: 5′ gaattcATGACGCGTAAACAGGCCACCAT and 5′ gtcgacTTACCCCTTGTTTGCAGCCCGGAA. After the digestion by EcoRI, this 1.17 kb DNA fragment was ligated with the EcoRI fragment of P*GAP/ENO* to form the *Z. mobilis* expressible P*GAP/ENO-metB* operon. The *Pfu-sHSP* gene was amplified from *P. furious* chromosomal DNA with the primer pair: 5′gaattcATGGTGAGGAGAATAAGAAGATG and 5′ gtcgacTTATTCAACTTTAACTTCGAATCCTTC. After the digestion by EcoRI, this 0.5 kb DNA fragments was ligated with the EcoRI fragment of P*GAP/ENO* to form the P*GAP/ENO-sHSP* operon. After the P*GAP/ENO-metB* operon was digested with SalI and the P*GAP/ENO-yfdZ* operon was digested with XhoI, two DNA fragments were ligated to get the P*GAP/ENO-metB/yfdZ* operon. After P*GAP/ENO- metB/yfdZ* operon was digested with XhoI and P*GAP/ENO-sHSP* operon was digested with SalI, two DNA fragments were ligated to result in the P*GAP/ENO-sHSP/metB/yfdZ* operon which could be cut out by SalI and XhoI (Figure 7).

**Figure 8:**
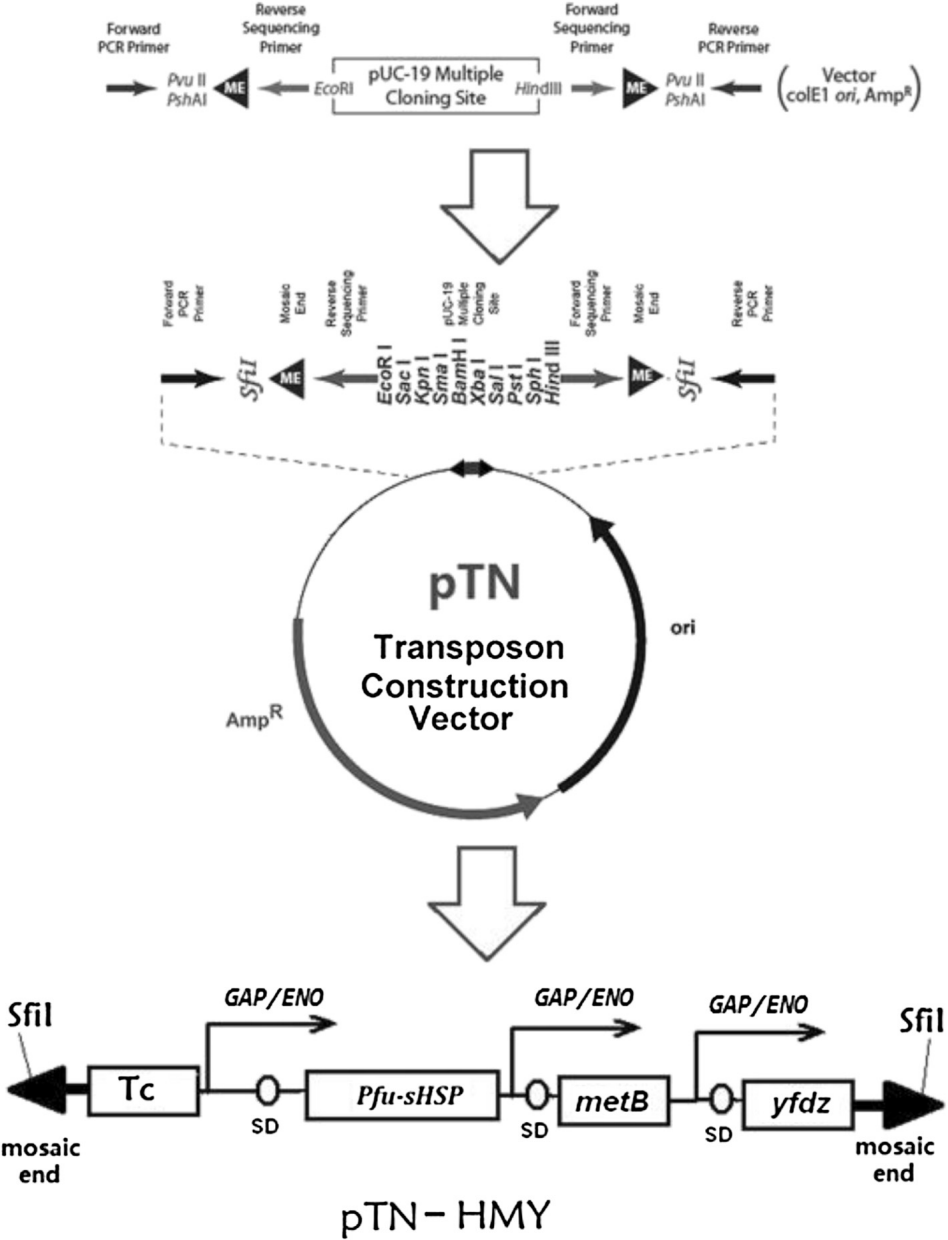
**Constructions of Tn5 transposon - pTN-HMY.** Commercial EZ-Tn5 pMOD vector was mutated to create vector pTN by substituting the PvuI and PshAI with SfiI restriction site. To allow selection of *Z. mobilis* transformants after electroporation with the Tn5 transposon, tetracycline resistance determinant (TC) was cloned into the multiple cloning site in the vector pTN. The *sHSP/metB/yfdZ* operon was cloned into the SalI site of pTN to create pTN-HYM. This plasmid contains SfiI restriction site recognized sequences flanking the mosaic end (ME) sites, which are specifically recognized by the EZ-Tn5 transposase.

The commercial EZ-Tn5 pMOD vector was modified by substituting the PvuI and PshAI restriction sites outside of both ends flanking Tn5 transposase recognition sequences with SfiI restriction site. The resulting plasmid was named as pTN, is a high-copy number, pUC19-based vector contains a multiple cloning site (MCS) between the hyperactive 19 bp Mosaic Ends (ME) that is specifically and uniquely recognized by Tn5 Transposase. Tn5-carrying plasmid pTN was modified further for integration of foreign DNA into *Z. mobilis*. The tetracycline resistance gene which was mutated without SalI restriction site (from the 1.4 kb BamHI fragment from p34S-Tc, Genebank: AF062082) was inserted into the BamHI site of plasmid pTN to result in the plasmid pTN-TC. The P*GAP/ENO-sHSP/metB/yfdZ* operon was obtained by digestion with SalI and XhoI, was inserted into the SalI site of pTN-TC. The resulting plasmid, designated as pTN-HMY (Figure 8), was then transformed into chemically competent *E. coli* Top10 cells (Invitrogen) and inoculated onto LB agar plates supplemented with tetracycline and ampicillin and sequence confirmed.

To prepare the transposome, the Tn5 transposon pTN-HYM was digested with SfiI. The 6.9 kb was purified from the agarose gel, and mixed with EZ-Tn5 transposase (Epicentre). The mixture was incubated for 30 min at room temperature, to allow the transposase to stably bind to the Tn5 Transposon DNA; that mixture was then stored at -20°C.

**Figure 9:**
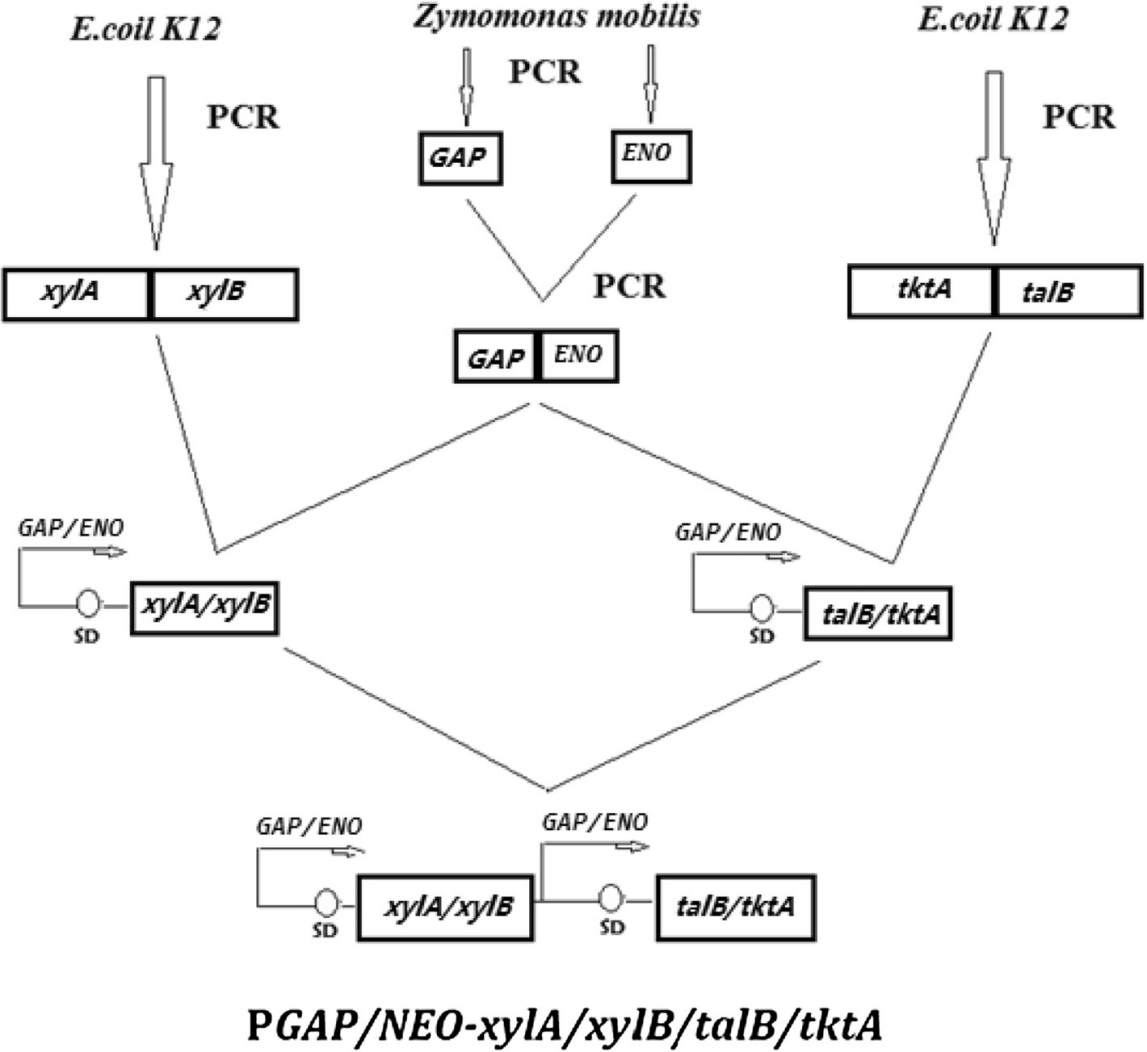
**Constructions of P*GAP/NEO*-*xylA/xylB/talB/tktA*.** A fusion promoter was cloned to facilitate the higher level expression of genes under its control*.* The *xylA/xylB* and the *talB/tktA* operons were used to add a metabolic pathway to utilize xylose to produce ethanol.

The *xylA/xylB* genes were amplified from *E. coli* K-12 chromosomal with the primer pair: 5′ gaattcATGCAAGCCTATTTTGACCAGC and 5′ gtcgacTTATTTGTCGAACAGATAATGGTTT. After the deletion of the SalI restriction site within the *xylA* gene by mutagenesis, this 2.87 kb DNA fragments were digested with EcoRI, and was ligated with the EcoRI fragment of P*GAP/ENO* to form the P*GAP/ENO*-xylA/xylB operon. The *talB/tktA* genes were amplified from *E. coli* K-12 chromosomal with the primer pair: 5′ gaattcATGACGGACAAATTGACCTCCCTTC and 5′ gtcgacTTACAGCAGTTCTTTTGCTTTC. After the deletion of the SalI restriction site within the *tktA* gene by mutagenesis, this 2.97 kb DNA fragments were digested with EcoRI, and was ligated with the EcoRI fragment of P*GAP/ENO* to form the *Z. mobilis* expressible P*GAP/ENO-talB/tktA* operon. After the P*GAP/ENO-xylA/xylB* operon were digested with XhoI and P*GAP/ENO-talB/tktA* operon was digested with SalI, two fragments were ligated to form the P*GAP/ENO-xylA/xylB/talB/tktA* operon which could be cut out by SalI and XhoI (Figure 9).

**Figure 10:**
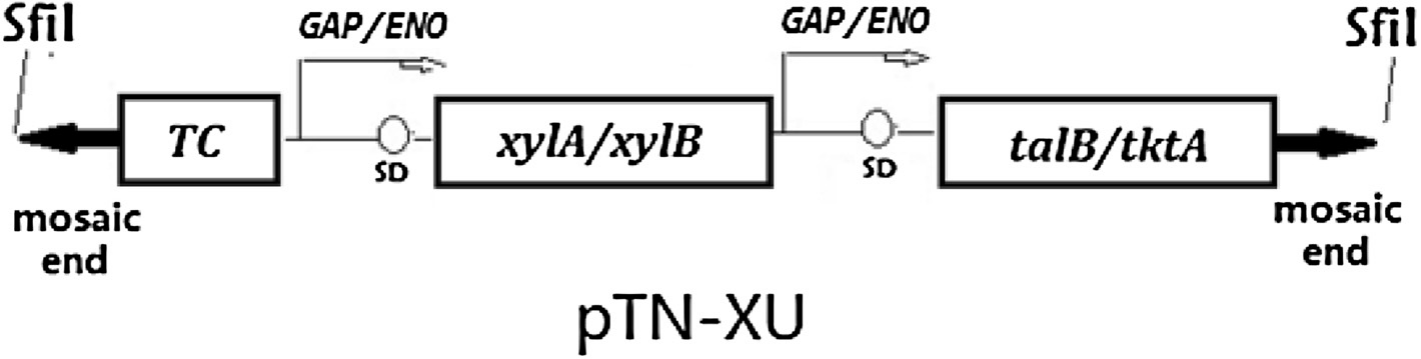
**Generation of Tn5 transposon vector pTN-XU for random mutagenesis of *Z. mobilis*.** The *xylA/xylB/talB/tktA* operon for adding a metabolic pathway for *Z. mobilis* to utilize xylose was inserted into the SalI site to create pTN-XU. This plasmid contains SfiI recognized sequences flanking the mosaic end (ME) sites, which are specifically recognized by the EZ-Tn5 transposase.

**Figure 11:**
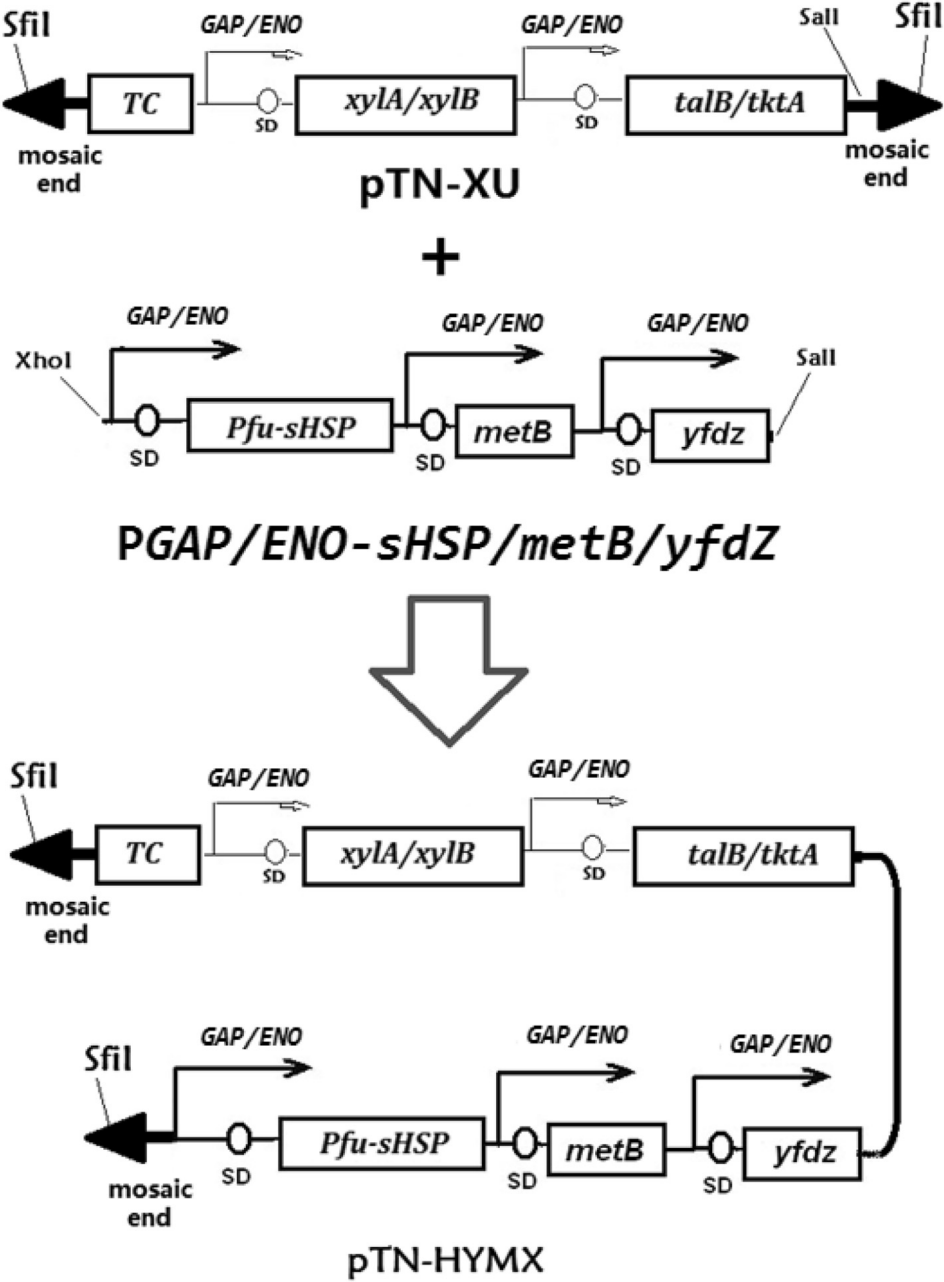
**Generation of Tn5 transposon vector pTN-HYMX for random mutagenesis of *Z. mobilis*.** The *xylA/xylB/talB/tktA* operon and *sHSP/metB/yfdZ* operons were cloned into the SalI site of pTN to create the plasmid pTN-HYMX. This plasmid contains SfiI recognized sequences flanking the mosaic end (ME) sites, which are specifically recognized by the EZ-Tn5 transposase.

After the digestion with SalI and XhoI, the P*GAP/ENO-xylA/xylB/talB/tktA* operon was recovered, and was inserted into the SalI site of pTN-TC. The resulting plasmid, named pTN-XU (Figure 10), was then transformed into chemically competent *E. coli* Top10 cells (Invitrogen) and inoculated onto LB agar plates supplemented with tetracycline or ampicillin. The whole P*GAP/ENO-sHSP/metB/yfdZ* operon was obtained by digestion with SalI and XhoI, was inserted into the SalI site of pTN-XU. The resulting plasmid, designated as pTN-HMYX (Figure 11).

After determining that *Z. mobilis CP4* was indeed sensitive to tetracycline, fresh electrocompetent cells were prepared by washing cells from 400 ml of a broth culture (RM medium) at an optical density at 600 nm (OD600) of 0.4 twice with equal volumes of ice-cold 10% glycerol. Cells were suspended again in 400 ul of washing buffer, and aliquots of 100 ul were dispensed into 0.2-mm gapped electroporation cuvettes along with 1 ul of pTN-HYM -Tn5 mixture. Electroporation was performed with a Bio-Rad Gene Pulser Xcell (6.0 ms, 1.5 kV). Cells were immediately diluted in 1 ml RM medium and plated onto RM agar supplemented with 30 ug/ml tetracycline.

After 2 days of incubation at 32°C, colonies on tetracycline-containing plates arose only when the Electroporation mixtures had contained pTN-HYM; not a single colony was observed in the mixtures without DNA. From three independent transformations, we picked 100 random colonies and those cells were used to inoculate in fresh RM liquid medium. To verify Tn5 integration, DNA was isolated using the Qiagen DNeasy blood and tissue kit, and Tn5 insertion within the genome were analyzed by PCR.

### Development of recombinant *Z. mobilis* with xylose utilization capability

Two kinds of transposome mixture (pTN-XU and pTN-HYMX) were electroporated into *CP4.* To prepare the transposome, pTN-XU or pTN-HYMX was digested with SfiI, so that the xylose-utilizing operon could be purified from an agarose gel, and mixed with EZ-Tn5 transposase (Epicentre). The mixture was incubated for 30 min at room temperature, to allow the transposase to bind stably to the Tn5 Transposon DNA; that mixture was then stored at -20°C.

Fresh *CP4* electrocompetent cells 100 ul were dispensed into 0.2-mm gapped electroporation cuvettes along with 1 ul of DNA-Tn5 transposase mixture. Electroporation was performed with a Bio-Rad Gene Pulser Xcell (6.0 ms, 1.5 kV). Cells were immediately diluted in 1 ml RM medium and plated onto agar supplemented with 50 ug/ml tetracycline and xylose as the only carbon source. After 2 days incubation at 32°C, colonies on tetracycline -containing plates arose only when the electroporation mixtures had contained DNA from pTN-XU or pTN-HYMX; not a single colony was observed in the mixtures without DNA. A total of about 6 × 10^2^ tetracycline (Tc)-resistant transformants were obtained in each of three independent transformation experiments with *CP4.* Colony from pTN-HYMX was designated as *Z. mobilis* HYMX (*HYMX*), and colony from pTN-XU designated as *Z. mobilis* XU (*XU*). From three independent transformations, we picked 50 random *HYMX* colonies and 50 random *XU* colonies. These cells were used to inoculate in fresh RM liquid medium. To verify DNA integration, *Z. mobilis* DNA was isolated using the Qiagen DNeasy blood and tissue kit, and were analyzed by PCR. PCR identification showed that, 92 from the above 100 strains had the associated genes been fully integrated into the genome. Strains with higher ethanol production were selected for further fermentation research.

## Competing interests

The authors declare that they have no competing interests.

## Authors’ contributions

HW and XJ set up and designed the study. WZ, TW and XJ cloned the plasmids and transposon; XZ, TW and WZ performed the characterization of selected strains. XZ, WZ and TW performed GC experiments; HW, XZ analyzed the data and wrote the manuscript. All authors discussed the results and commented on the manuscript, and all authors read and approved the final manuscript.
